# Deep learning-based automated lesion segmentation on pediatric focal cortical dysplasia II preoperative MRI: a reliable approach

**DOI:** 10.1186/s13244-024-01635-6

**Published:** 2024-03-13

**Authors:** Siqi Zhang, Yijiang Zhuang, Yi Luo, Fengjun Zhu, Wen Zhao, Hongwu Zeng

**Affiliations:** 1 Shantou University Medical College, Shantou University, 22 Xinling Road, Jinping District, Shantou, 515041 China; 2 Department of Radiology, Shenzhen Children’s Hospital, District, 7019 Yitian Road, Futian, Shenzhen, 518038 China; 3 Department of Epilepsy Surgical Department, Shenzhen Children’s Hospital, 7019 Yitian Road, Futian District, Shenzhen, 518038 China

**Keywords:** Malformations of cortical development, Refractory epilepsy, Pediatrics, Deep learning, Magnetic resonance imaging

## Abstract

**Objectives:**

Focal cortical dysplasia (FCD) represents one of the most common causes of refractory epilepsy in children. Deep learning demonstrates great power in tissue discrimination by analyzing MRI data. A prediction model was built and verified using 3D full-resolution nnU-Net for automatic lesion detection and segmentation of children with FCD II.

**Methods:**

High-resolution brain MRI structure data from 65 patients, confirmed with FCD II by pathology, were retrospectively studied. Experienced neuroradiologists segmented and labeled the lesions as the ground truth. Also, we used 3D full-resolution nnU-Net to segment lesions automatically, generating detection maps. The algorithm was trained using fivefold cross-validation, with data partitioned into training (*N* = 200) and testing (*N* = 15). To evaluate performance, detection maps were compared to expert manual labels. The Dice-Sørensen coefficient (DSC) and sensitivity were used to assess the algorithm performance.

**Results:**

The 3D nnU-Net showed a good performance for FCD lesion detection at the voxel level, with a sensitivity of 0.73. The best segmentation model achieved a mean DSC score of 0.57 on the testing dataset.

**Conclusion:**

This pilot study confirmed that 3D full-resolution nnU-Net can automatically segment FCD lesions with reliable outcomes. This provides a novel approach to FCD lesion detection.

**Critical relevance statement:**

Our fully automatic models could process the 3D T1-MPRAGE data and segment FCD II lesions with reliable outcomes.

**Key points:**

• Simplified image processing promotes the DL model implemented in clinical practice.

• The histopathological confirmed lesion masks enhance the clinical credibility of the AI model.

• The voxel-level evaluation metrics benefit lesion detection and clinical decisions.

**Graphical Abstract:**

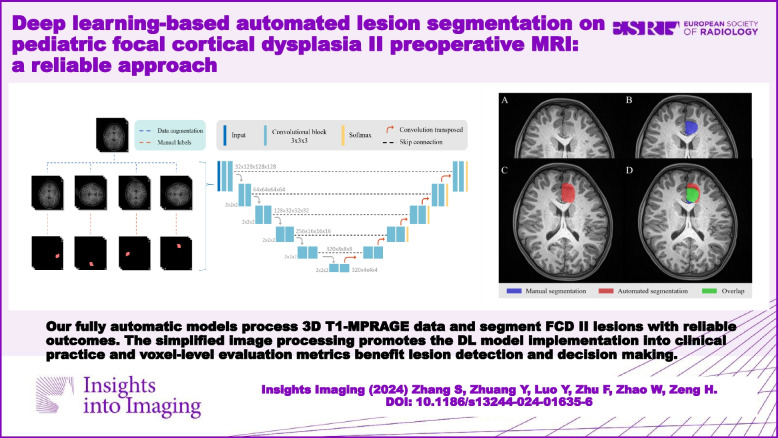

**Supplementary Information:**

The online version contains supplementary material available at 10.1186/s13244-024-01635-6.

## Introduction

Focal cortical dysplasia (FCD) is defined as a localized malformation of cortical development caused by disturbances in neural cell proliferation, migration, and differentiation [1]. It is the most common cause of refractory epilepsy in children, accounting for more than 30% [2]. In 2011, the International League Against Epilepsy (ILAE) classified FCD into three types according to histopathological features. Among FCD patients treated with surgical therapy, about 29–39% were type II [3]. The final strategy for drug-resistant focal epilepsy is surgical resection. The outcome is increasingly encouraging, with 70% of patients achieving seizure freedom [4].

**Fig. 1:**
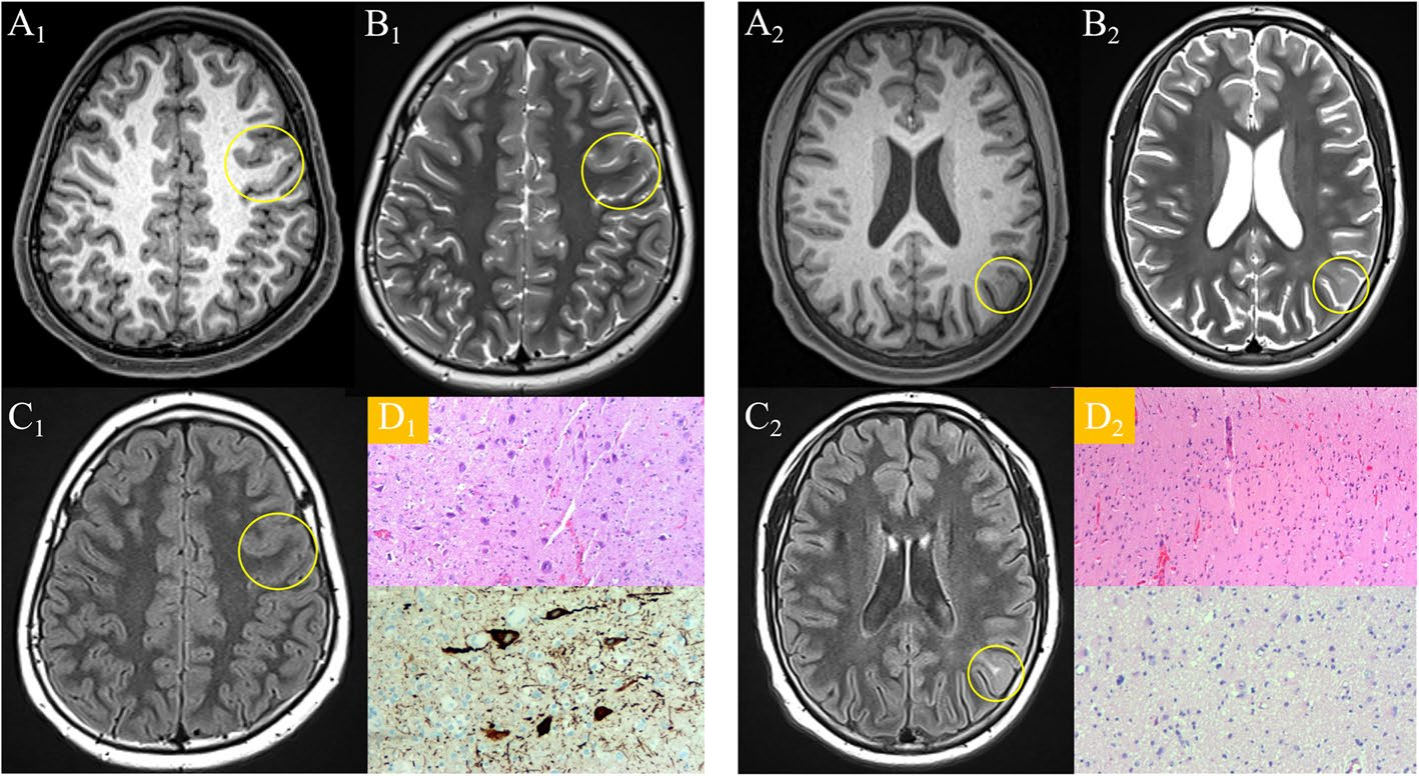
Representative structure neuroimaging findings of FCD II. **A**_**1**_**–D**_**1**_ The imaging and histopathology data of a 12-year-old male patient who was diagnosed with FCD IIa. Preoperative 3D T1-MPRAGE imaging revealed localized cortical thickening and blurred gray-white matter boundary (encircled) in the left precentral gyrus. **A**_**2**_**–D**_**2**_ The imaging and histopathology data of a 15-year-old male patient who was diagnosed with FCD IIb. Preoperative FLAIR imaging demonstrated a hyperintense lesion extending into the lateral ventricle (transmantle sign, circled) in the left parietal lobe

Accurate pre-surgical lesion localization was the key impact factor for the outcome [5]. Three-dimension high-resolution structure MRI has become mandatory. The detailed MRI signs include cortex thickening, gray-white matter blurring, transmantle sign, and signal intensity changes in both the gray and white matter [6]. The FCD II had typical MRI features (examples shown in Fig. 1). Ordinarily, experienced neuro-radiologists can make a correct diagnosis and portray the whole lesion accurately. However, the reality is that very minimal abnormalities are reflected in subtle MRI signal alteration, which is beyond the limitations of the human eye to detect. This discrepancy is the leading cause of postoperative seizure recurrence [6].

Artificial intelligence has entered a new era and scientists have placed considerable effort into improving the detection of FCD lesions, establishing many computer-assisted approaches [7]. Some semi-automated computational post-processing methods, such as voxel-based morphometry (VBM) [8] and surface-based morphometry (SBM) [9], use statistical methods to find areas of the brain that differ from normal controls. Martin et al. [8] demonstrated the strengths and limitations of different VBM approaches in epilepsy imaging and found that VBM based on T2-FLAIR had the best specificity and junction map had the best sensitivity. Unfortunately, only 5% of their MRI-negative patients had a histopathological proven FCD result, meaning that the reliability of model performance in the FCD cohort was indeed to be improved.

Further, recent advances in convolutional neural networks are at the forefront of image detection and segmentation tasks [10]. Neural network architectures designed for segmentation, such as U-Nets, attained remarkable achievements within the pertinent domains, especially radiology [11] and pathology [12]. These algorithms enable automated optimal feature extraction, which has paved the way for learning more essential features than any manual feature extraction-based methods [13]. The new approaches based on machine learning (ML) and deep learning (DL) also dramatically influenced the field of automatic FCD detection in MRI-negative focal epilepsies [14–17].

There are still several challenges in translating computer vision tools into clinical applications. First, the heterogeneity caused by differences in MRI scanners, sequences, and field strengths may affect the morphological and intensity feature values. Second, the standard of predicted lesion clusters and the filtering criteria for false positives were inconsistent [9, 14], which hampers the widespread use of morphometric measurements. Third, the different levels (voxel-, vertex-, lesion-, or patient-level) of lesion detection outputs restrict the model evaluation and comparison. Finally, the use of standard brain templates distorts the lesion and normalized processing limits the boundaries of abnormal brain regions as well.

To solve the above problems, we conduct a fully automated method for lesion detection and segmentation with the minimum input, routine clinical FCD II pre-surgical 3D T1-weighted magnetization-prepared rapid gradient-echo (MPRAGE) images. We present a 3D full-resolution nnU-Net architecture, the advance of which combined the U-Net architecture with data preprocessing techniques to improve efficiency and simplify application [18]. With the voxel-level lesion detection outputs, our networks could assist epilepsy surgeons in implementing visible and effective preoperative evaluation.

## Method

### Patients

**Fig. 2:**
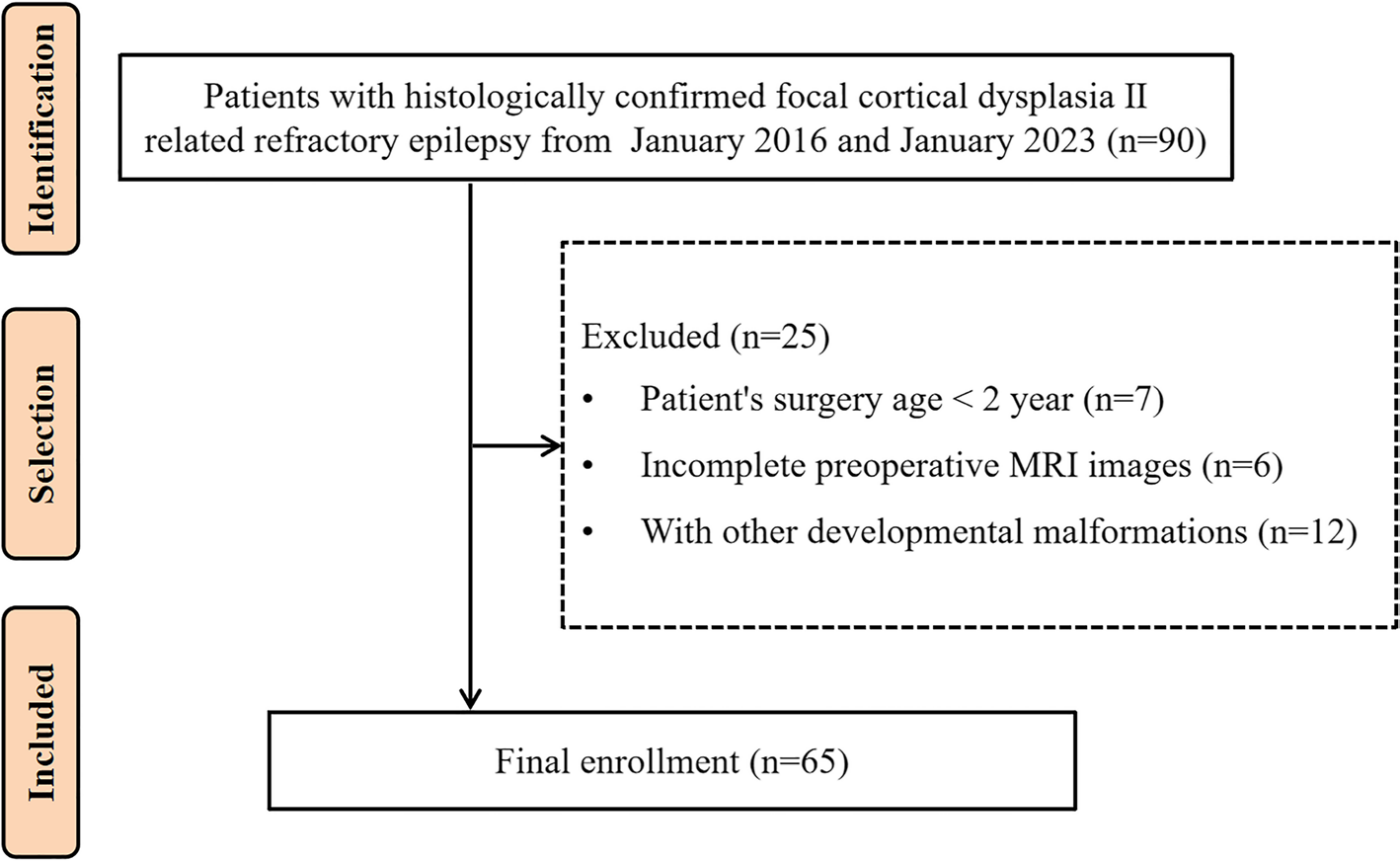
Flowchart of patient selection

Clinical and radiology data of refractory epilepsy patients, confirmed with a pathological diagnosis of FCD II, were retrospectively reviewed and analyzed. All patients were from Shenzhen Children’s Hospital, which is a tertiary epilepsy center and the only pediatric center in southern China. These patients were hospitalized between January 2016 and January 2023. Refractory epilepsy was defined as follows according to Clinical Diagnosis and Treatment Guidelines: Volume of Epilepsy (2015 Revised Edition). Refractory epilepsy is when the seizures are still not completely controlled after a sufficient amount and sufficient course of reasonable treatment with two or more antiseizure medications. The inclusion criteria included the following: (1) the age of the patient at the time of epilepsy surgery was between 2 and 18 years; (2) baseline clinical data were available and complete; (3) both the pre- and post-surgical brain three-dimensional high-resolution MRI data were collected. Exclusion criteria were unqualified preoperative MRI images or combined with other developmental malformations (such as tuberous sclerosis, hemispherical dysplasia, and periventricular nodular heterotopia). Sixty-five cases were finally included in this study. This study was approved by the local institutional review board. Figure 2 shows the workflow for patient selection.

### Image acquisition and labeling

MRI data of all patients were acquired before surgery using a 3.0-T scanner (Skyra, Siemens, Germany). The 3D T1-MPRAGE [19] parameters were as follows: TR = 2000 ms, TE = 2.4 ms, slice thickness = 1 mm, slice spacing = 0, slice number = 176, matrix size = 256 × 256.

Referencing a successful experience for a relatively small size of dataset from Stanford Artificial Intelligence Lab, the rotation technique of data enhancement was employed before training the deep learning model [20]. The training dataset was augmented using an augmentation technique defined through a suitable function class named Volumentations 3D, which is implemented in Python. It is available via https://github.com/ZFTurbo/volumentations. The dataset was divided into two sets for training and testing, containing 50 and 15 cases, respectively. Using the data augmentation techniques, the number of training samples was increased to 200 cases.

The surgically resected area, including the histopathologically confirmed FCD II lesion, was used as a gold standard to define the ground truth of FCD [21]. The mask was manually drawn using ITK-SNAP software (version 3.8.0) in collaboration between two experienced neuroradiologists, each with over three years of expertise in epilepsy imaging, ensuring a singular representation of a lesion per subject. One rater created each lesion mask, subsequently reviewed by the other. In instances of uncertainty regarding the ROI extension, both clinical data and postoperative MRI were considered until a consensus was reached by both reviewers. In complicated cases without agreement between the two neuroradiologists, a third senior pediatric neuroradiologist provided the final opinion to establish consensus. Manual labels served as target parameters for the training of the DL model.

### Automatic detection framework

**Fig. 3:**
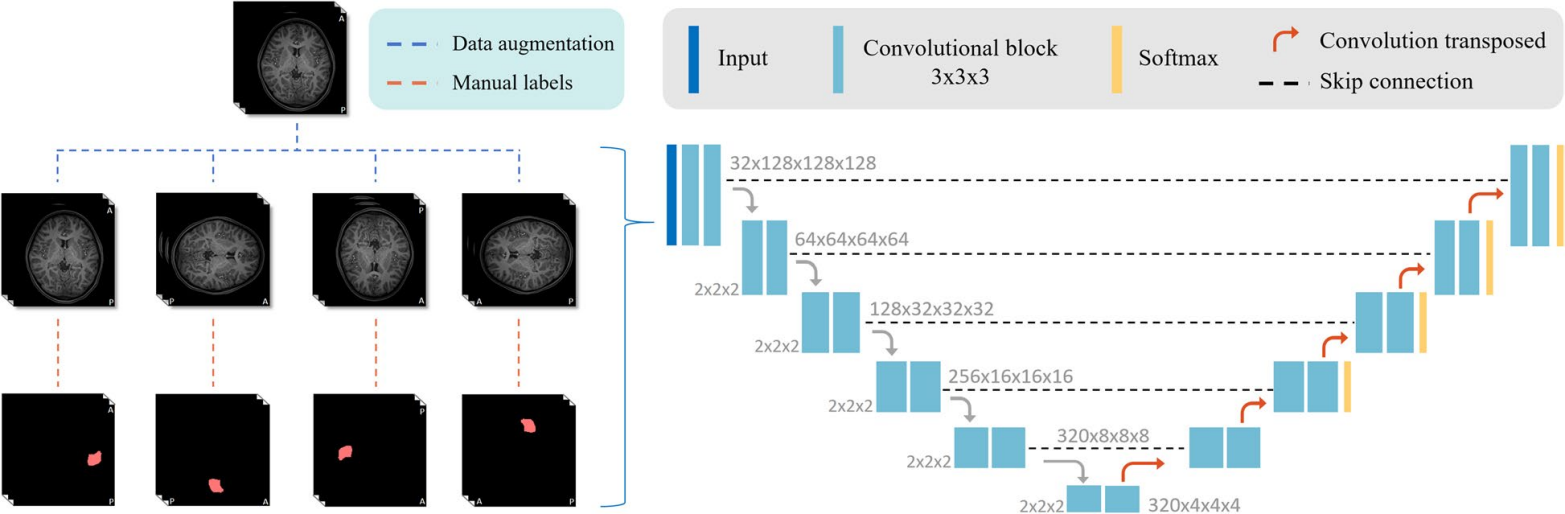
Flowchart of automatic detection network

This study uses a segmentation-oriented approach for automatic FCD II detection and localization, where each voxel in the image is assigned either a lesional or non-lesional label. The models in the proposed pipeline were developed using the self-configuring framework for medical segmentation, nnU-Net [18]. The 3D architecture was generated by the nnU-Net with its default parameters. The 3D T1-MPRAGE images were then used as input channels to the network, together with the corresponding manual segmentation (MS). The training process of the nnU-Net was performed using a fivefold cross-validation. At the inference time, the trained network was used to generate automated segmentations (AS) in the testing cohort. A schematic representation of the inference pipeline from the original image input to the final lesion segmentation is shown in Fig. 3.

### Evaluation metrics

On a per-voxel basis, true-positive (TP), true-negative (TN), false-positive (FP), and false-negative (FN) values of the AS compared to the ground truth (MS) were assessed. The Dice similarity coefficient (DSC) was calculated to measure the overlap between the manual and automated segmentation, as well as the AS sensitivity [22]. The sensitivity reflects the probability for a voxel to be included in the AS when present in the ground truth.

### Statistical analysis

SPSS 18.0 (IBM, New York, USA) statistical analysis software was used for data analysis. Continuous variables are described as means (standard deviation, SD) or medians (interquartile range, IQR) and categorical variables are presented as frequencies (%).

## Results

### Clinical characteristics

The demographic and clinical characteristics of the patients are summarized in Table 1. A total of 65 patients were included in the present study, including 36 males and 29 females. The mean age of the patients at epilepsy surgery was 6.38 years (SD, 4.77). Of the 65 patients who underwent epilepsy surgery, 44 had focal seizures, and 30 had lesions located in the frontal lobe. According to the ILAE, 30 people with epilepsy (46%) were classified as FCD IIa and 35 (54%) as FCD IIb. MRI was considered negative in six patients (12%) in the training cohort vs. two patients (13%) in the testing cohort.

**Table 1:** Clinical characteristics of the patients in two datasets

	**Training set (*n* = 50)**	**Testing set (*n* = 15)**
Age at surgery (years), mean (SD)	6.3 (4.6)	6.7 (5.3)
Male sex, *n* (%)	30 (60)	6 (40)
Seizure types, *n* (%)		
Focal seizures	34 (68)	10 (67)
Secondarily generalized seizures	16 (32)	5 (33)
Lesion location, *n* (%)		
Frontal lobe	24 (48)	6 (40)
Non-frontal lobe	26 (52)	9 (60)
Histology, *n* (%)		
FCD IIa	23 (46)	7 (47)
FCD IIb	27 (54)	8 (53)
MRI-negative	6 (12)	2 (13)

### Model performances

**Fig. 4:**
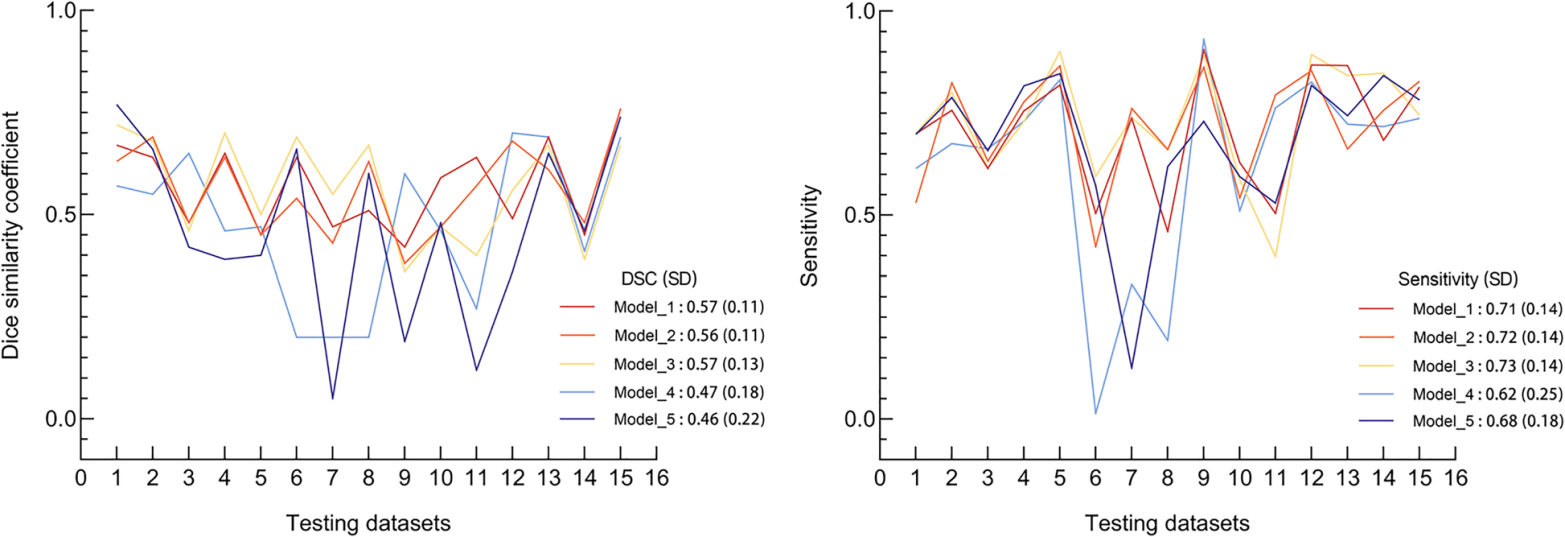
Comparison across different models and input image modalities from 15 testing datasets based on Dice similarity coefficient (DSC) and sensitivity

**Fig. 5:**
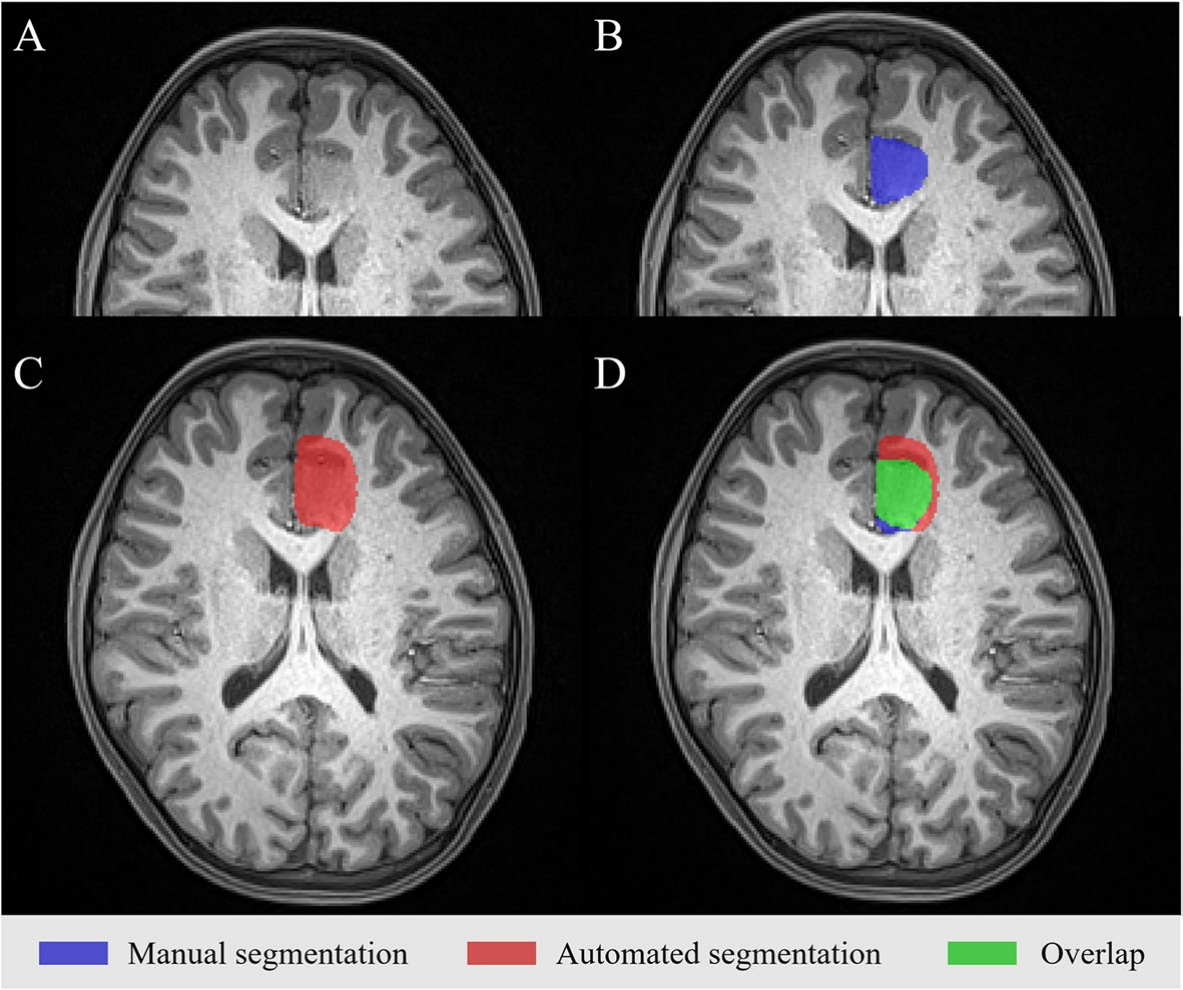
The example of inference results: images of a 9-year-old boy, who was diagnosed with FCD IIb. **A** The raw images as input. **B** The manual segmentation as labels. **C** The automated segmentation outcomes inference by Model_3. **D** The overlap zone of MS and AS. The mean DSC score and the mean sensitivity value of the patient were 0.69 and 0.72, respectively

The performances of the five different FCD II detection network configurations on the internal fivefold cross-validation sets are shown in Table 2 and Fig. 4. Evaluation against the testing dataset of the trained nnU-Net resulted in a median number of the detection lesions for each model was 5 (IQR = 2–6), 3 (IQR = 2–5), 3 (IQR = 2–5), 5 (IQR = 3–6), and 6 (IQR = 4–7), respectively. Regarding lesion segmentation performance, Model_3 achieved the best performance, with a mean DSC score of 0.57 and a mean sensitivity value of 0.73. The automated lesion segmentation visualization results of a patient in the test dataset are shown in Fig. 5 as an example.

**Table 2:** The performances of the five models

	**Mean DSC (SD)**	**Mean sensitivity (SD)**
Model 1	0.57 (0.11)	0.71 (0.14)
Model 2	0.56 (0.11)	0.72 (0.14)
Model 3	0.57 (0.13)	0.73 (0.14)
Model 4	0.47 (0.18)	0.62 (0.25)
Model 5	0.46 (0.22)	0.68 (0.18)

## Discussion

As we know, preoperative lesion detection is the key to the success of surgery, which is the major assignment of radiologists. Nevertheless, artificial intelligence is gradually revolutionizing this job [23]. Conventionally, for refractory epilepsy brain MRI, neuroradiologists inevitably miss subtle lesions which can be detected by neural networks. Efforts to enhance the detection of FCD primarily center around three key facets: sequence improvement, morphological analysis, and model optimization.

Many works only use T1WI sequences [24, 25] since this sequence allows for optimal evaluation of brain anatomy and morphology [19]. Due to the presence of local hyperintensities in some FCD cases, some detection models [14, 15] added FLAIR sequences to improve model performance. In addition, Flaus et al. [26] proposed a deep learning-based PET image enhancement method using simulated PET to improve lesion visualization, from 38 to 75%, in a 37-case adult cohort. Although the combination of multiple imaging techniques would benefit the subtle FCD detection [27], we aim to simplify input requirements. To make our approach more usable for children’s examination, we ensured a robust detection of FCD II using the simplest imaging modality, 3D T1-MPRAGE images, without requiring manual intervention due to error-prone preprocessing steps, such as cortical surface reconstruction. Moreover, cutting down scan time could also help reduce the anxiety of children and parents, which can enhance image quality.

Cortical thickening is one of the typical FCD radiographic features [6]; accordingly, morphometric technique was widely used for automatic segmentation. With seven years of clinical usage experience, Sepulveda et al. [25] demonstrated that applying FreeSurfer software (one of the semiautomated automated brain segmentation methods) could increase detection sensitivity, especially in cases with the absence of clear conventional MRI findings. A recent multicenter, multinational study presented an interpretable, fully automated pipeline for surface-based detection of FCD, which reached a sensitivity of 67% in the test cohort [9]. Progress is also being made on automated volumetric MRI methods. Martin et al. [8] and Wang et al. [28] reported a detection rate of 65% for VBM postprocessing in the MRI-negative group, while Martin et al. [8] and Wagner et al. [29] showed a detection rate of 85% with morphometric analysis program in the MRI-positive patients. Nevertheless, the studies using the VBM method detected lesions at the patient level, which was insufficient to determine the lesion’s borders. Although the features obtained by the SBM method based on the multi-dimensional analysis of the lesion cortex were accurate to the lesion level, inconsistencies remained in the criteria for predicting lesion clusters and filtering out false positives [14, 15]. The evaluation metrics of our work were on a voxel level, which would benefit lesion detection and clinical decisions. The sensitivity value of the best model to detect lesions in the test cohort was 0.73, which was comparable to the previous works.

In recent years, several neural networks have been proposed for detecting and segmenting FCDs using AI. Mo et al. [30] extracted cortical surface features and then classified them with the artificial neural network. Thomas et al. [13] proposed a Multi-Res-Attention Unet, a hybrid skip connection-based convolutional neural network architecture for FCD segmentation. David et al. [24] created a feed-forward artificial neural network for FCD detection based on the morphometric output maps of MAP18. Feng et al. [16, 17] used Bayesian classifiers trained on different numbers of feature maps to detect FCD in FLAIR-negative MRIs. Gill et al. [14] provided a multicenter validation study of the detection of MRI-negative FCD using an uncertainty-informed Bayesian deep learning algorithm as a measure of confidence for risk stratification. House et al. [15] developed a 3D convolutional neural network with autoencoder regularization for FCD detection and segmentation and validated it prospectively on daily routine MRIs. The nnU-Net framework is a plug-and-play framework for deep learning-based biomedical segmentation that automatically configures itself for each new task [18]. The nnU-Net has been applied in various tumor segmentation, including pancreatic ductal adenocarcinoma [31], osteosarcoma [32], breast cancer [33], and so on. We achieved broadly similar results concerning the Dice score with other studies [13–15], the mean DSC score of the best model to detect lesions in our testing cohort was 0.57.

This study has several potential limitations. First, it has limitations inherent in the single-center study design and the use of augmentation techniques. Second, given that FCD II is the most prevalent epileptogenic developmental brain malformation and a common cause of surgically treatable epilepsy, our study exclusively concentrates on this subtype. The FCD I datasets would be included in future research to enhance the clinical applicability of our models. Additionally, due to the inherent differences in neuroimaging features between FCD IIa and IIb, we conducted the independent sample *t*-test to assess potential variations in the model’s lesion detection performance across these two subtypes. The results suggest that, concerning the evaluation metrics of DCS and sensitivity, although the models exhibited superior performance in detecting lesions in group IIb compared to group IIa, the observed disparity between the two groups did not reach statistical significance. Further details can be found in Supplementary Table 1. Apart from that, age is a crucial factor influencing the quality of T1-MPRAGE images. To address this concern and account for ongoing myelination, we excluded children under 2 years old at the time of surgery. The acquisition of MRI data was meticulously performed within the week preceding the surgery, aiming to minimize potential age-related interference with image quality. Finally, due to the lack of independent external validation datasets, the possibility of model overfitting cannot be ruled out. These issues need to be further explored in future work.

## Conclusions

This pilot study confirmed that 3D full-resolution nnU-Net can automatically segment FCD lesions with reliable outcomes. The proposed models achieve a maximum DSC score of 0.57 and the highest sensitivity value of 0.73 in the testing datasets. These promising results inspire us to conduct additional validation with multi-center datasets as this technique progresses towards use in clinical practice.

### Supplementary Information

**Additional file 1: Supplementary Table 1.** The performances of the five models in two different pathological subtypes.

## Data Availability

The datasets used and/or analyzed during the current study are available from the corresponding author upon reasonable request.

## Abbreviations

AS: Automated segmentations
DL: Deep learning
DSC: Dice-Sørensen coefficient
FCD: Focal cortical dysplasia
FN: False negative
FP: False positive
ILAE: International League Against Epilepsy
IQR: Interquartile range
ML: Machine learning
MPRAGE: Magnetization-prepared rapid gradient-echo
MS: Manual segmentation
SBM: Surface-based morphometry
SD: Standard deviation
TN: True negative
TP: True positive
VBM: Voxel-based morphometry
