# Supplementary material for: Deep learning-based automated lesion segmentation on pediatric focal cortical dysplasia II preoperative MRI: a reliable approach
Source: Insights Imaging. 2024 Mar 13;15:71. doi: 10.1186/s13244-024-01635-6 (PMC10933224; doi:10.1186/s13244-024-01635-6)
Supplement: Supplementary file 1 — Additional file 1: Supplementary Table 1. The performances of the five models in two different pathological subtypes. [file 13244_2024_1635_MOESM1_ESM.pdf]

# Deep learning-based automated lesion segmentation on pediatric focal cortical dysplasia II preoperative MRI: a reliable approach

## ELECTRONIC SUPPLEMENTARY MATERIAL

Supplementary Table 1. The performances of the five models in two different pathological subtypes.

| | FCD Ⅱ a (n = 7) | FCD Ⅱ b (n = 8) | *t* value | P value |
|---|---|---|---|---|
| **Mean DSC (SD)** | | | | |
| Model 1 | 0.56 (0.09) | 0.58 (0.13) | -0.186 | 0.855 |
| Model 2 | 0.54 (0.09) | 0.58 (0.13) | -0.632 | 0.538 |
| Model 3 | 0.54 (0.13) | 0.59 (0.13) | -0.725 | 0.482 |
| Model 4 | 0.40 (0.19) | 0.54 (0.16) | -1.499 | 0.158 |
| Model 5 | 0.41 (0.26) | 0.51 (0.19) | -0.791 | 0.443 |
| **Mean sensitivity (SD)** | | | | |
| Model 1 | 0.66 (0.13) | 0.75 (0.15) | -1.227 | 0.241 |
| Model 2 | 0.67 (0.16) | 0.76 (0.10) | -1.382 | 0.199 |
| Model 3 | 0.68 (0.17) | 0.77 (0.10) | -1.301 | 0.216 |
| Model 4 | 0.54 (0.29) | 0.69 (0.22) | -1.125 | 0.281 |
| Model 5 | 0.60 (0.24) | 0.75 (0.08) | -1.689 | 0.115 |
